# Genome-wide identification, classification and transcriptional analysis of nitrate and ammonium transporters in *Coffea*

**DOI:** 10.1590/1678-4685-GMB-2016-0041

**Published:** 2017-04-10

**Authors:** Tiago Benedito dos Santos, Joni Esrom Lima, Mariane Silva Felicio, João Danillo Moura Soares, Douglas Silva Domingues

**Affiliations:** 1 Laboratório de Biotecnologia Vegetal, Instituto Agronômico do Paraná, Londrina, PR, Brazil; 2 Programa de pós-graduação em Agronomia, Universidade do Oeste Paulista (UNOESTE), Presidente Prudente, SP, Brazil; 3 Departamento de Botânica, Instituto de Ciências Biológicas, Universidade Federal de Minas Gerais (UFMG), Belo Horizonte, MG, Brazil; 4 Centro de Energia Nuclear na Agricultura (CENA), Escola Superior de Agricultura “Luiz de Queiroz” (ESALQ), Universidade de São Paulo (USP), Piracicaba. SP, Brazil; 5 Departamento de Botânica, Instituto de Biociências de Rio Claro, Universidade Estadual Paulista “Júlio Mesquita Filho” (UNESP), Rio Claro, SP, Brazil

**Keywords:** Coffee, nitrogen transport, nitrogen uptake, gene family evolution

## Abstract

Nitrogen (N) is quantitatively the main nutrient required by coffee plants, with acquisition mainly by the roots and mostly exported to coffee beans. Nitrate (NO_3_^–^) and ammonium (NH_4_^+^) are the most important inorganic sources for N uptake. Several N transporters encoded by different gene families mediate the uptake of these compounds. They have an important role in source preference for N uptake in the root system. In this study, we performed a genome-wide analysis, including *in silico* expression and phylogenetic analyses of *AMT1*, *AMT2*, *NRT1*/PTR, and *NRT2* transporters in the recently sequenced *Coffea canephora* genome. We analyzed the expression of six selected transporters in *Coffea arabica* roots submitted to N deficiency. N source preference was also analyzed in *C. arabica* using isotopes. *C. canephora* N transporters follow the patterns observed for most eudicots, where each member of the *AMT* and *NRT* families has a particular role in N mobilization, and where some of these are modulated by N deficiency. Despite the prevalence of putative nitrate transporters in the *Coffea* genome, ammonium was the preferential inorganic N source for N-starved *C. arabica* roots. This data provides an important basis for fundamental and applied studies to depict molecular mechanisms involved in N uptake in coffee trees.

## Introduction

Nitrogen (N) is one of the primary macronutrients and is a critical nutrient for plant growth and development (Konishi and Yanagisawa, 2014). N is imported into the roots through specific ion transporters in root cells from several sources. The main N inorganic forms absorbed by plants are ammonium (NH_4_^+^) and nitrate (NO_3_^–^). NO_3_^–^, due to nitrification reactions in the soil, is usually present in higher concentrations and is more mobile in soil when compared to NH_4_^+^ (Marschner, 2012; Luo *et al*., 2013). Nonetheless, both ions can be utilized by plants, NH_4_^+^ and NO_3_^–^ have different energetic and biochemical characteristics for assimilation, resulting in different net fluxes of both ions and NH_4_^+^ - NO_3_^–^ preference in plants (Patterson *et al*., 2010; Alber *et al*., 2012). These preferences are still poorly understood in tropical woody dicots.

Plants have evolved different transport systems that effectively adapt to changes of N availability in the environment. Ammonium and nitrate in plants have two uptake systems: a low-affinity transport system (LATS) operating in the millimolar concentration range and a saturable high-affinity transport system (HATS) operating at submillimolar concentrations (reviewed in Forde, 2000; Wang *et al*., 2012). Mainly LATS accomplish the N uptake at high external concentrations, while at concentrations below 0.5 mM N, uptake is achieved through HATS.

Nitrate uptake by plant roots from soil solution is mediated by members of four gene families: *NRT1*/PTR (NPF, nitrate transporter 1/peptide transporter family), *NRT2*, CLC (chloride channels), and SLAC1/SLAH (slow anion channel-associated 1 homologues) (Dechorgnat *et al*., 2011; Wang *et al*., 2012; Léran *et al*., 2014). The largest families in *Arabidopsis* are *NRT1* (involved in LATS) and *NRT2* (involved in HATS), with 53 and 7 copies, respectively. Proteins of the AMT/Rh/Mep family (Ludewig *et al*., 2007) mediate ammonium transport across membranes. Both *NRT*s and *AMT*s are variable in their biochemical properties, tissue localization and transcriptional regulation.

AMT1s and AMT2s usually contain 11 putative transmembrane domains (Couturier *et al*., 2007; McDonald *et al*., 2012). The members of the *AMT1* family are responsible for high affinity NH_4_^+^ transport (von Wirén *et al*., 2000; Yuan *et al*., 2007), whereas at least some plant *AMT2* members seem to transfer net NH_3_, yet no ionic currents across the membrane (Guether *et al*., 2009). The physiological roles of the *AMT2* proteins are less well understood than those of *AMT1* proteins (Neuhäuser *et al*., 2009).

AMTs and NRTs have been characterized in several plant species and genera: *Citrus* (Camañes *et al*., 2009), *Arabidopsis thaliana* (Wang *et al*., 2012; Xu *et al*., 2012), *Solanum lycopersicon* (Graff *et al*., 2011), *Medicago truncatula* (Young *et al*., 2011; Pellizzaro *et al*., 2014), *Cucumis sativus* (Migocka *et al*., 2013), *Zea mays* (Sorgona *et al*., 2011), *Sorghum bicolor* (Koegel *et al*., 2013) and *Puccinellia tenuiflora* (Bu *et al*., 2013). However, there are no reports on the molecular mechanisms of N uptake in coffee, including preferential N source.

Coffee is one of the most traded commodities in the world, and Brazil has the largest production and is the second largest consumer of the beverage (Lashermes *et al*., 2008; Mondego *et al*., 2011). Fertilization practices are among the most important costs in coffee production (Fehr *et al*., 2012). The genus *Coffea* (Rubiaceae) has 124 species (Davis *et al*., 2011), with *Coffea arabica* and *C. canephora* being the two species of greatest economic importance (Vidal *et al*., 2010). *C*. *arabica* is an allotetraploid (2n = 4x = 44 - C^a^C^a^E^a^E^a^) and *C. canephora* is a diploid species (2n = 2x = 22 - CC), allogamous and self-incompatible (Denoeud *et al*., 2014). *C. arabica* originated from a spontaneous hybridization between two diploid coffee species, *C. canephora* and *C. eugenioides* (2n = 2x = 22 - EE) (Vidal *et al*., 2010).

We present here a phylogenetic reconstruction of *AMT1*, *AMT2*, *NRT1*/PTR, and *NRT2* gene families from the recently released *Coffea canephora* genome (Denoeud *et al*., 2014). These phylogenies are supplemented with transmembrane domain and subcellular localization predictions, and *in silico* expression profiling in *C. canephora* organs. We have also investigated the transcriptional responses of selected transporters under N starvation in *C. arabica*, as well as identified preferential N sources for uptake in *C. arabica* roots under N starvation. This study provides the basis to develop future in-depth physiological and molecular studies to fully address N utilization in plants of the *Coffea* genus, and opens a perspective on the understanding of modules that control NH_4_^+^ and NO_3_^–^ homeostasis in coffee roots, which are important targets for breeding and biotechnology.

## Material and Methods

### Identification and phylogenetic analysis of NRT and AMT gene families in coffee

Basic procedures of annotation followed a report on the evolution of nitrate and ammonium transporters (von Wittgenstein *et al*., 2014). Protein sequences of AMTs and NRTs annotated in *Arabidopsis thaliana*, *Medicago truncatula*, *Populus trichocarpa* and *Vitis vinifera* by von Wittgenstein *et al*. (2014) were used as queries for BLASTP searches against the *C. canephora* genome (http://coffee-genome.org/).

The parameters BLASTP used were also based on von Wittgenstein *et al*. (2014), with an expected threshold lower than 1e-50 and at least 30% of identity. Transmembrane (TM) domains were predicted using TMHMM v2 software (Krogh *et al*., 2001). Sequences with at least 8 TM domains were compared with the reference sequences, and only the ones that had a maximum difference of 50 amino acids in length were selected for further analyses. Subcellular localization was predicted using MultiLoc2 (Blum *et al*., 2009), with the MultiLoc2-HighRes (Plant), 10 Locations algorithm. For phylogenetic analyses, we included *Oryza sativa, Zea mays, Sorghum bicolor* and *Brachypodium distachyon* protein sequences for each transporter family. Sequences were aligned using MUSCLE (Edgar, 2004). This alignment was used to generate neighbor-joining trees (Saitou and Nei, 1987) based on distance matrices using the Jones-Taylor-Thornton model and pairwise deletion. The resampling method was bootstrapping and consisted of 1,000 replicates. All procedures were run using MEGA6 software (Tamura *et al*., 2013). Phylogenies were rooted using *Arabidopsis* sequences belonging to another family as outgroup.

### Transcriptional profile of N transporters in *C. canephora*

For *in silico* expression profiling, RNAseq data from different organs and tissues of *C. canephora* were obtained from the “RNA-seq RPKM” track available on JBrowser at the Coffee Genome Hub database (http://coffee-genome.org/; Dereeper *et al*., 2015). This data was compiled to a spreadsheet to generate heatmaps that use a color coding to differentiate expression levels. The expression unit used was reads per kilobase per million reads (RPKM). The software Bio-Analytic Resource for Plant Biology (BAR) HeatMapper Plus (http://bar.utoronto.ca/) was used to generate the heatmaps of AMTs and NRTs genes.

### Transcriptional profile of N transporters in *C. arabica* roots - N starvation experiment

We evaluated the transcriptional profile of 3 AMT and 3 NRT transporters in *C. arabica* roots submitted to N starvation (Table 1), which had homologs in ESTs of the Brazilian Coffee Genome Consortium database (Mondego *et al*., 2011). Basic procedures of N starvation experiment are summarized in Figure S1 (Supplementary material). Overall procedures and plant nutritive solution are detailed in de Carvalho *et al*. (2013). After 4 weeks on hydroponic devices for acclimation, *C. arabica* L. cv. IAPAR59 5-month old plants were harvested for time point 0 and then transferred to a modified N-free solution, where lateral roots were harvested at 1 day and 10 days after transfer into the N-free solution. Experiments were conducted twice, with a minimum of three biological replicates per experiment. All samples were harvested between 09:30 am and 10:30 am. Biological replicates were represented by pools of coffee lateral roots of at least nine plants each, at the same developmental stage. After harvesting, all samples were frozen immediately in liquid nitrogen and stored at −80 °C until RNA extraction.

**Table 1:** *Coffea arabica* transcriptional analysis: RT-PCR primers and orthologs in *Arabidopsis* and *C. canephora* genomes.

Gene	Forward Primer	Reverse Primer	NCBI Accession	Orthologs	
				*Arabidopsis thaliana*	*Coffea canephora*
*CaAMTa*	AGCCGAATACATCTGCAACC	GAAGGTATGTGGTGTCGATGG	GW473095	AT4G13510	Cc03_g06810
*CaAMTb*	CATTCCTTCGGGCTCTTACA	GCAATGGAGCCACTGGTTAT	GW483639	AT4G13510	Cc01_g14140
*CaAMTc*	TGCGTGCATTGTATCTTCTGA	GCAGTCCATGGAGAAGAAGC	GT683246	AT2G38290	Cc07_g19360
*CaNRTa*	TATGCCTTGGTGTCATTGGA	CTGCTGCAGACACCTTGAAA	GW479551	AT1G69850	Cc02_g36020
*CaNRTb*	CTCGGAGAGAAAGATGAGCAG	GGACCCAACCACCAGTTTTA	GW442751	AT2G26690	Cc06_g08580
*CaNRTc*	GCTGCTGCTGTGGAAGAAGT	CCAAGCTTCTCAAAGGTCTCA	GT693501	AT5G62680	Cc04_g15770

### RNA isolation, cDNA synthesis and semi-quantitative RT-PCR

Total RNA from *C*. *arabica* L. cv. IAPAR59 roots was isolated following the same procedures used by previous studies of our group (dos Santos *et al*., 2011). We treated RNA samples with DNase to remove traces of DNA contamination, and after dissolved in RNase-free water, the RNA concentration was determined using a NanoDrop® ND-100 spectrophotometer (Waltham, MA, USA). Complementary DNA (cDNA) was synthesized in a final volume of 20 μL using 5 μg of total RNA by using SuperScript® III Reverse Transcriptase (Invitrogen), following the manufacturer's instructions

Primers (Table 1) were designed using Primer Express (version 3.0) according to parameters established by the software to obtain amplicons of 100 base pairs with a Tm of 60 °C ± 1 °C (Table 1). Amplification was performed according to the following temperature profile: 2 min initial denaturation at 94 °C; 30 cycles of 94 °C for 1 min, 60 °C for 1 min, 72 °C for 1 min; end extension of 3 min at 72 °C; final hold at 4 °C. Cycles for RT-PCR analysis were based on Brandalise *et al*. (2009). Amplicons were verified in 2% agarose gel electrophoresis with sodium boric acid (SB) buffer (0.5 M NaOH, pH adjusted to 8.5 with boric acid), stained with ethidium bromide and photographed. The captured images were processed for densitometric analysis using the ImageJ 1.43 U software, as previously described by Freschi *et al*. (2009) and dos Santos *et al*. (2015). Transcriptional profiles were normalized using *EF1*α, a reference gene recommended by de Carvalho *et al*. (2013) for this condition. Semi-quantitative RT-PCR analysis was repeated at least three times for each sample.

### Measurement of ^15^N influx in coffee roots

*Coffea arabica* L. cv. IAPAR59 seedlings were hydroponically grown under non-sterile conditions in a greenhouse according to the following regime: 14/10 h light/dark and temperature 28 °C/18 °C. Plants were grown in nutrient solution containing 1 mM KH_2_PO_4_, 1 mM MgSO_4_, 250 μM K_2_SO_4_, 250 μM CaCl_2_, 100 μM Na-Fe-EDTA, 50 μM KCl, 50 μM H_3_BO_3_, 5 μM MnSO_4_, 1 μM ZnSO_4_, 1 μM CuSO_4_, and 1 μM NaMoO4 (pH adjusted by 2 mM MES, pH 5.8, Sigma-Aldrich). The nutrient solution was replaced every two days during the first week. After the acclimation period, the plants were submitted to N sufficient (+N, 2 mM NH_4_NO_3_) or N starvation (-N, without N supply) nutrient solution for three days. Influx measurements of ^15^N-isotope in plant roots were conducted after rinsing the roots in 1 mM CaSO_4_solution for 1 min, followed by an incubation for 10 min in nutrient solution containing 0.2 mM or 2 mM of ^15^N-isotope with the equimolar concentration containing either ^15^NH_4_NO_3_ (42.52 atom% ^15^N) or NH_4_^15^NO_3_ (41.62 atom% ^15^N) as a sole N source, and finally washed in 1 mM CaSO_4_solution. Roots were harvested and stored at −70 °C before milled. Each sample was ground in liquid N_2_ and dried at 55 °C for five days. The ^15^N and %N determination was performed by isotope ratio mass spectrometry (ANCA SL da Sercon, England) with 5 mg of dried samples.

### Statistical Analysis

Statistical analyses was done by one-way ANOVA using Sisvar software (Ferreira, 2011), followed by Tukey's multiple comparison tests (p < 0.05 level).

## Results and Discussion

### Genome-wide analysis of ammonium transporters in *C. canephora* genome

**Figure 1:**
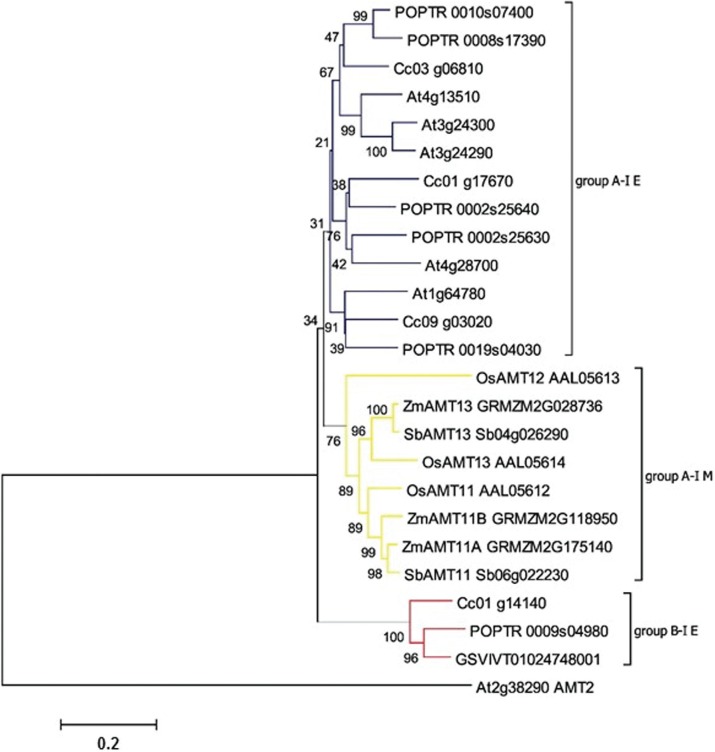
Neighbor joining phylogenetic analysis of the *AMT1* family. The tree was rooted using an *A. thaliana AMT2* gene as an outgroup. Percent bootstrap values from 1,000 replicates are given. All *C. canephora* genes are placed in clades with > 50% of bootstrap support. Taxonomic groups are colored based on groups: blue refers to group A eudicot sequences; yellow represents monocot sequences in group A, and red illustrates dicot sequences in group B. Accession numbers are shown. Codes were retrieved from the Coffee Genome Hub for *C. canephora* and Phytozome for all other species. Phylogenetic groups were based in von Wittgenstein *et al*. (2014).

All N transporter families in *C. canephora* had a copy number under the range found for most eudicots (von Wittgenstein *et al*., 2014; Pii *et al*., 2014; Table 2). We identified eight copies of ammonium transporter genes, four belonging to the *AMT1* family and the others to *AMT2*. Three members of super-group A compose the *C. canephora AMT1* family, and one member belongs to B super-group (Figure 1), which is highly contrastant to *Populus*, a tree that contains an expanded family of ammonium transporters (Couturier *et al*., 2007; von Wittgenstein *et al*., 2014). The presence of only one member in the coffee tree genome suggests that genome duplication mechanisms were not relevant to *AMT1* evolution in coffee trees.

**Table 2:** Comparison of members from the *AMT1*, *AMT2*, *NRT1*/PTR and *NRT2* gene families in Viridiplantae, based in von Wittgenstein *et al*. (2014) and Pii *et al*. (2014).

	Number of members			
Species	AMT1	AMT2	NRT1/PTR	NRT2
*Arabidopsis thaliana*	5	1	51	6
*Coffea canephora*	4	4	57	3
*Glycine max*	5	5	96	3
*Oryza sativa*	2	6	65	3
*Physcomitrella patens*	5	10	18	8
*Populus trichocarpa*	6	5	70	7
*Selaginella moellendorfii*	1	0	31	2
*Setaria italica*	2	6	74	7
*Vitis vinifera*	1	1	44	4

The *AMT1* gene family comprises four members with 9-11 predicted TM domains. Almost all *AMT1* transporters were predicted to be located in the endoplasmic reticulum, and only one *AMT1* (*Cc03_g06810*) has been indicated with subcellular localization in the Golgi apparatus. Members of *AMT2* family have 11 TM domains and only one (*Cc07_g11400*, sub-group B-II-E) was localized on the Golgi apparatus. Other *C. canephora AMT2* members (sub-group A-II-E and B-I-E) are located in the endoplasmic reticulum. Interestingly, we did not find any *AMT2* transporter in *C. canephora* from super-group A-I, the sole group with biochemically characterized members (von Wittgenstein *et al*., 2014). Detailed information on TM prediction and subcellular localization are available in Tables S1 and S2.

*In silico* expression analysis of putative *C. canephora AMT1* genes (Figure 3A) showed *Cc01_g14140* as the lowest expressed *AMT1* gene. Two genes (*Cc01_g17670* and *Cc09_g03020*) were preferentially transcribed in roots, whereas *Cc03_g06810* had higher expression in aboveground organs. *Cc01_g17670* is the ortholog of the *AtAMT1;4* gene (*At4g28700*), with 75% of identity. Both genes belong to group A-I E (Figure 1). *AtAMT1;4* is a pollen high-affinity ammonium transporter; the overexpression of this gene in roots of mutant plants demonstrated that this gene is able to mediate ammonium uptake into *A. thaliana* roots (Yuan *et al*., 2009). Thus, probably *Cc01_g17670* is also a high-affinity ammonium transporter, but not specific to pollen as *AtAMT1;4*, due to the low expression in stamina (0,1 RPKM).

*Cc03_g06810*, the ortholog of the *AtAMT1;1* gene of *A. thaliana* (*At4g13510*), was the only gene expressed in all tissues, with higher expression in perisperm, indicating that ammonium transport may have some impact in fruit development. Compared to other *Arabidopsis AMT1* genes, *AtAMT1;1* is expressed more broadly, including roots, sepals, and leaves (von Wittgenstein *et al*., 2014), which may also explain the expression of *Cc03_g06810* in all *C. canephora* tissues.

**Figure 2:**
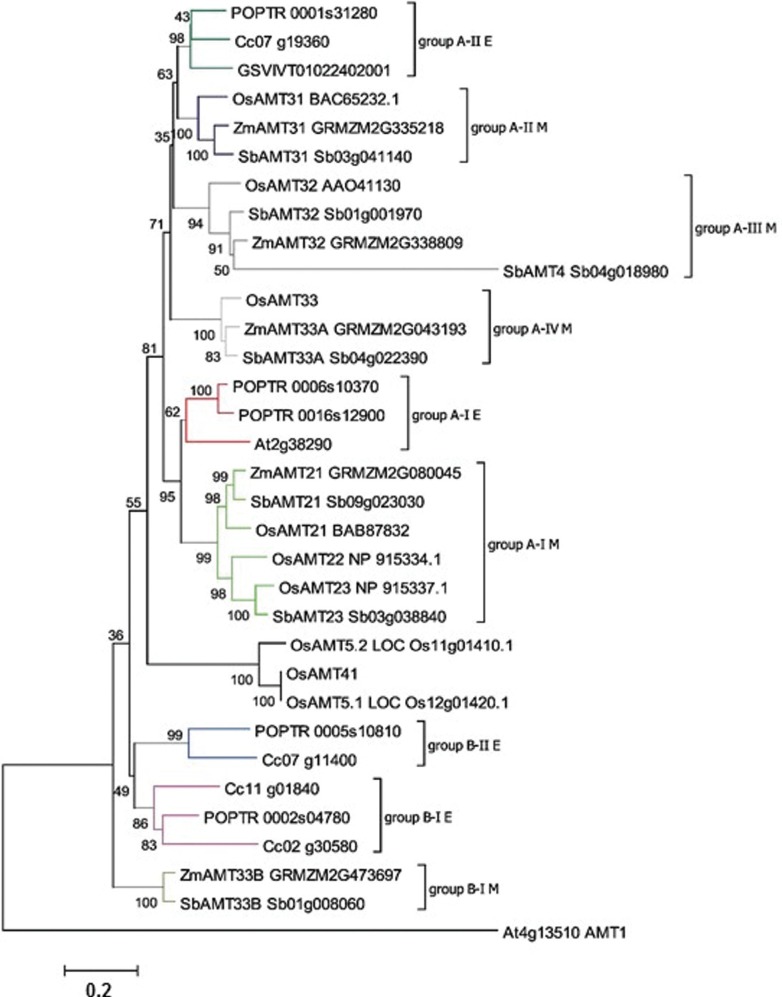
Neighbor joining phylogenetic analysis of the *AMT2* family. The tree was rooted using an *A. thaliana AMT1* gene as an outgroup. Percent bootstrap values from 1,000 replicates are given. All *C. canephora* genes are placed in clades with > 50% of bootstrap support. Taxonomic groups are colored based on groups: dark green and red refer to group A eudicot sequences; dark blue, dark grey, light grey and light green represent monocot sequences in group A-II; blue and purple illustrate dicot sequences in group B, grey represents group B monocot sequences. Accession numbers are shown. Codes were retrieved from the Coffee Genome Hub for *C. canephora* sequences and Phytozome for all other species. Phylogenetic groups were based in von Wittgenstein *et al*. (2014).

**Figure 3:**
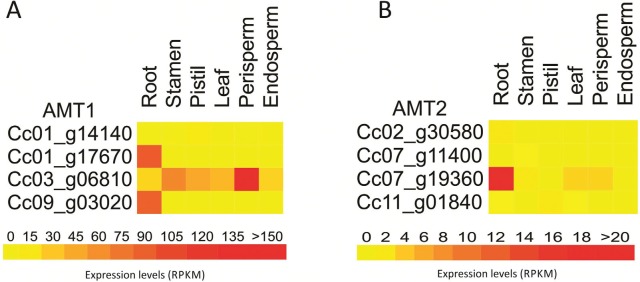
*In silico* expression profile of *C. canephora AMT1* (A) and *AMT2* (B) gene families. RNAseq data from roots, stamen, pistil, leaves, perisperm, and endosperm were obtained from Coffee Genome Hub database.

Considering a RPKM > 1, two members of the *AMT2* family had expression in roots and two in aerial parts (Table S2). *Cc07_g19360* was the highest expressed *AMT2* gene, with prevalence in roots (Figure 3B). The closest homolog in *Populus*, *POPTR_0001s31280*, (Figure 2) has a proeminent expression in reproductive tissues (von Wittgenstein *et al*., 2014), a pattern that is not observed in coffee.

### Nitrate transporters in the *C. canephora* genome

**Figure 4:**
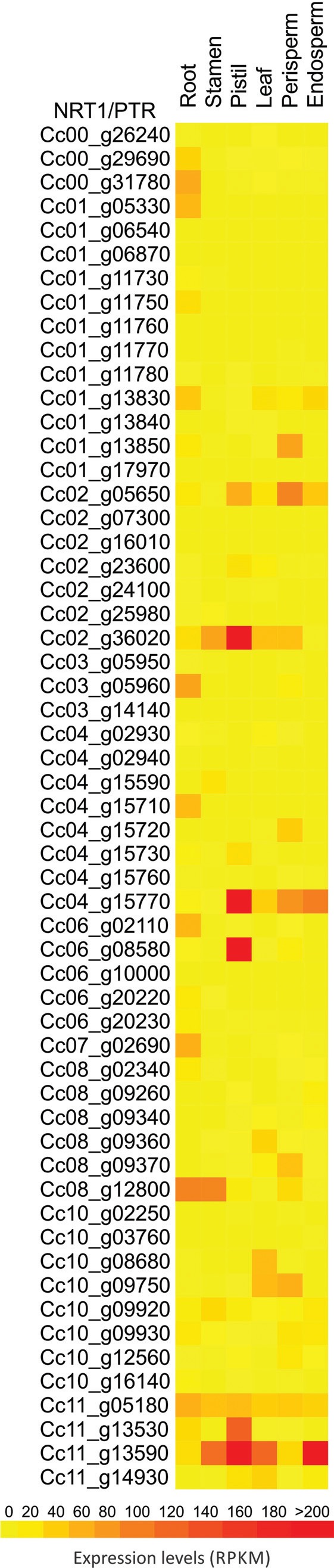
*In silico* expression profile of *C. canephora NRT1/PTR* gene family. RNAseq data from roots, stamen, pistil, leaves, perisperm, and endosperm were obtained from Coffee Genome Hub database.

The *NRT1*/PTR and *NRT2* families were represented by 57 and three copies, respectively, in the *C. canephora* genome (Table 2). The *C. canephora NRT1*/PTR family is similar to the average for land plants, which has 54 family members (von Wittgenstein *et al*., 2014). Most of them were predicted as cytoplasmatic (25) or located in the plasma membrane (15) (Table S3). Other genes were located in Golgi apparatus (12), peroxisome (3), endoplasmic reticulum (*Cc01_g06540*) and extracellular regions (*Cc0_g31780*). The members of this family possess from 8-12 predicted TM domains. All 10 *NRT1* superfamilies are represented in *C. canephora* (Figure S2). Considering an RPKM > 1, 12 *NRT1*/PTR members were expressed in all tissues and three genes (*Cc01_g11750, Cc04_g15710, Cc01_g05330*) were exclusively expressed in roots (Figure 4, Table S3).

*Cc08_g12800*, although expressed in aboveground organs, was the transcriptionally most active NRT transporter in roots (Figure 4, Table S3). The *Arabidopsis* ortholog of this gene is *AtNRT1;1* (*At1g12110*); both genes are in super-group B, group I E (Figure S2). *AtNRT1;1* is highly expressed in roots, and is described as a dual transporter that acts in high and low-affinity nitrate uptake, mediated by phosphorylation (Liu and Tsay, 2003). Phylogenetic relations and expression profiles indicate that *Cc08_g12800* probably has the same function as *AtNRT1;1*.

We observed that the gene *Cc11_g13590* is the most expressed in aboveground organs (Table S3). This gene is in the super-group D, group IV E with the ortholog *AtNRT1;7* (*At1g69860*) (Figure S2), that is expressed in phloem of older leaves, more specifically restricted to the sieve element and companion cell complex (Fan *et al*., 2009). Therefore, the probable function of this gene is to transport nitrate from older leaves to tissues demanding N (Fan *et al*., 2009). If the *Cc11_g13590* gene shares the same function of its ortholog, this may be the reason for the higher expression of this gene in several tissues. In pistils, the most expressed gene of *NRT1*/PTR family was *Cc04_g15770*, whose ortholog in the *Arabidopsis* genome is *AtNPF2.11* (*At5g62680*), also named as *AtGTR2*. This gene acts as a transporter for glucosinolates, suggesting that it possibly evolved through neo-functionalization of *NRT1*/PTR family members (Nour-Eldin *et al*., 2012). *AtNPF2.11* has its higher transcription in petals, sepals, and stamina (von Wittgenstein *et al*., 2014). Since *Cc04_g15770* had almost no expression in stamina, more studies are necessary to characterize the role of that *C. canephora* gene in reproductive tissues. In the seed and perisperm, the gene that had higher transcriptional values was *Cc02_g05650*, in agreement with the high expression profile of its ortholog oligopeptide transporter *At2g40460* in *Arabidopsis* seeds (von Wittgenstein *et al*., 2014). These two genes are part of the super-group I, group I E (Figure S2).

**Figure 5:**
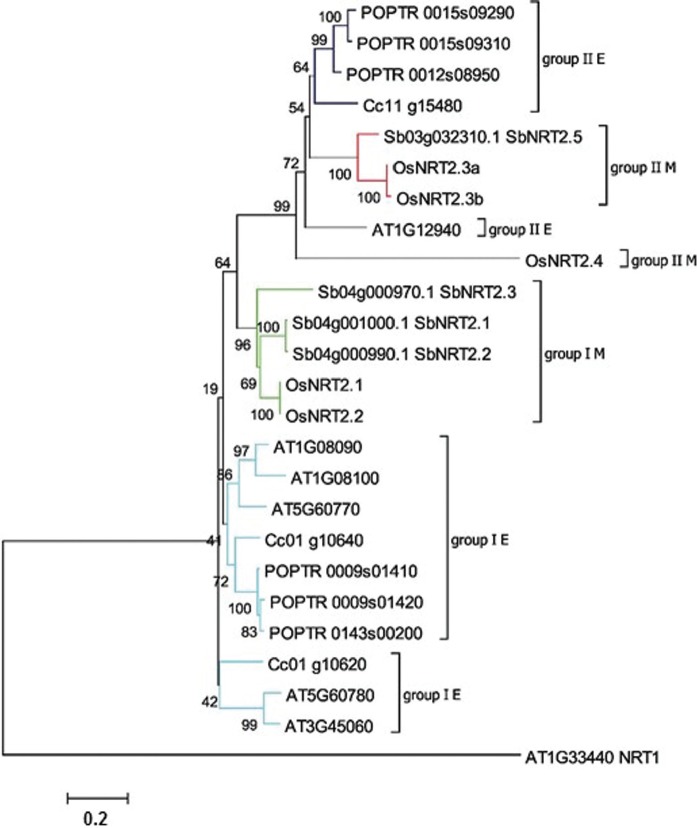
Neighbor joining phylogenetic analysis of the *NRT2* family. The tree was rooted using an *A. thaliana NRT1* gene as an outgroup. Percent bootstrap values from 1,000 replicates are given. Taxonomic groups are colored based on groups: light blue to group I eudicot sequences; green represent monocot sequences in group I; dark blue and grey illustrate dicot sequences in group II; red and grey represent group B monocot sequences. Accession numbers are shown. Codes were retrieved from the Coffee Genome Hub for *C. canephora* and Phytozome for all other species. Phylogenetic groups were based in von Wittgenstein *et al*. (2014).

**Figure 6:**
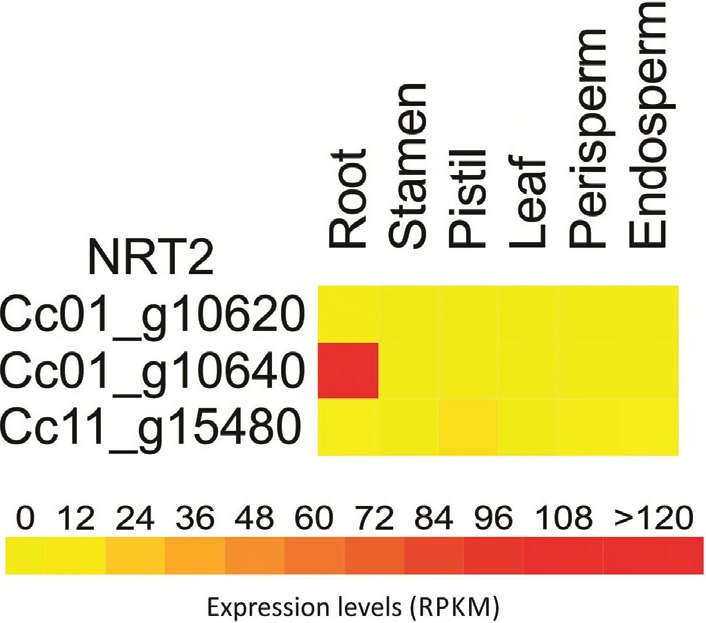
*In silico* expression profile of *C. canephora NRT2* gene family. RNAseq data from roots, stamen, pistil, leaves, perisperm, and endosperm were obtained from Coffee Genome Hub database.

The *NRT2* family comprises three members (Figure 5) that were predicted with 11 TM domains, two of them predicted to be localized in peroxisome, whereas the other one (*Cc01_g10620*) was predicted to be in cytoplasm. The *NRT2* gene family had two genes exclusively expressed in roots, considering RPKM > 1 (Table S4), where the most active was *Cc01_g10640*. The gene with higher expression in the aerial parts (*Cc11_g15480*) was also the less expressed in roots (Figure 6; Table S4).

*AtNRT2;1* (*At1g08090*) shares higher identity with *Cc01_g10640* than the other *Arabidopsis NRT2* genes in group I E (Figure 5). *AtNRT2;1* is the major inducible high-affinity transporter of nitrate (iHATS). When this gene was disrupted in *Arabidopsis*, 72% of the iHATS was reduced (Li *et al*., 2007). The transcriptional profile of *Cc01_g10640* suggests that this gene probably acts in the same function as *AtNRT2;1*. *AtNRT2;1* is targeted to the root plasma membrane (Chopin *et al*., 2007), but the predicted localization of *Cc01_g10640* is in peroxisomes. According to von Wittgenstein *et al*. (2014), the high degree of peroxisome localization for *NRT2* is unexpected, and it may be due to difficulties predicting hydrophobic, membrane bound proteins, added to inaccuracy in recently-released genome annotations.

The gene *Cc11_g15480*, that has been shown more expressed in aerial parts is related to *AtNRT2;5* (*At1g12940*), and these genes are in group II E. *AtNRT2;5* is highly expressed in senescing leaves, and is described as being a nitrate repressible gene, having maximum expression in the absence of nitrate (Okamoto *et al*., 2003).

### Transcriptional responses of N transporters in *C. arabica* roots

We analyzed the transcriptional profile of three AMT and three NRT transporters in *C. arabica* in response to N depletion. Orthologs of these genes in *C. canephora* genome are indicated in Table 1 and Supplementary Tables S1 to S3. Since transcriptional changes related to the lack of N sources can also be species-specific, it is important to highlight that further work should address if the same transcriptional pattern is observed in *C. canephora*; but, to our knowledge, this is the first study that evaluates the transcriptional profile of N transporters in coffee tree roots.

**Figure 7:**
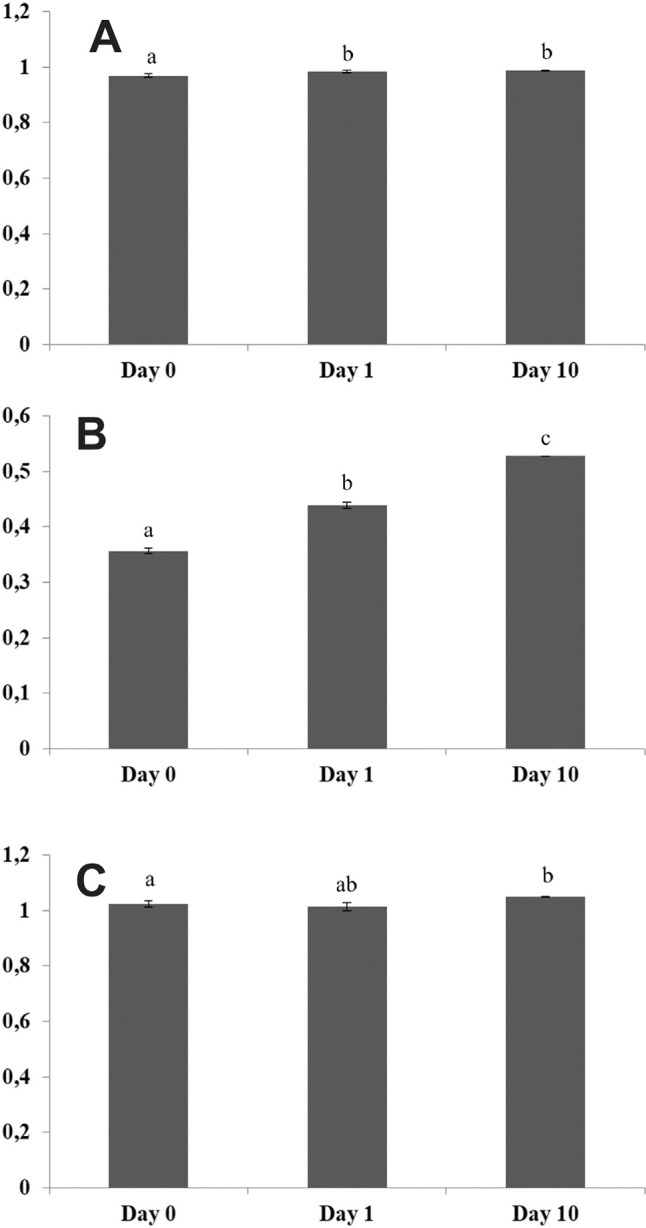
Densitometric analysis of semi-quantitative RT-PCR (Figure S3) for *CaAMTa* (A), *CaAMTb* (B) and *CaAMTc* (C) using ImageJ 1.43 U software. *EF1*α gene was used as an internal control to normalize the expression level. The data represent the mean ± standard deviation of three biological replicates. Letters indicate significant differences between genotypes in each treatment by Tukey test (p < 0,05).

*CaAMTa* and *CaAMTb* were induced by N starvation (Figure 7A and B). *CaAMTb* is an *AMT1* transporter with low expression in roots of *C. canephora* (Figure 3, Table S1), which is in agreement with RT-PCR analysis, where this gene was the lesser expressed among the ammonium transporters under N-sufficient conditions. Under N suppression, *CaAMTb* was the most induced gene, suggesting its participation in ammonium uptake in N-deficiency conditions and warranting further studies in functional analyses to depict its transport capacity.

**Figure 8:**
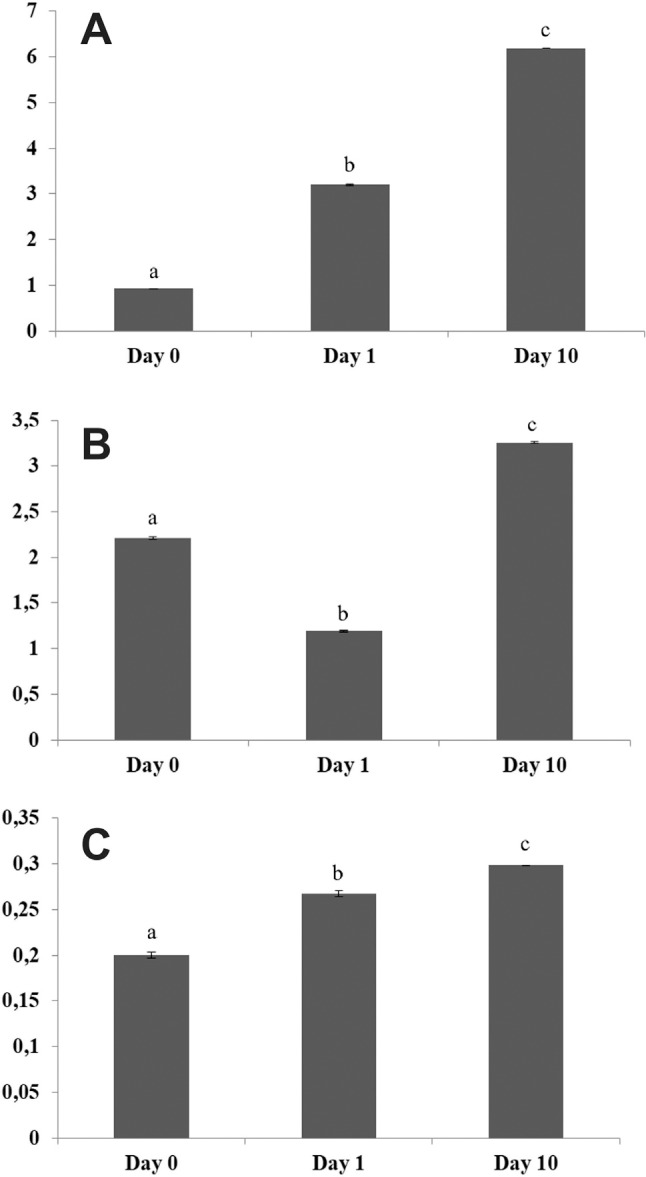
Densitometric analysis of semi-quantitative RT-PCR (Figure S3) for *CaNRTa* (A), *CaNRTb* (B) and *CaNRTc* (C) using ImageJ 1.43 U software. *EF1*α gene was used as an internal control to normalize the expression level. The data represent the mean ± standard deviation of three biological replicates. Letters indicate significant differences between genotypes in each treatment by Tukey test (p < 0,05).

Nitrate transporters displayed distinct transcriptional patterns (Figure 8): *CaNRTa* and *CaNRTc* showed an increasing gradient of transcripts, suggesting a direct role in molecular responses to N starvation, while *CaNRTb* was down-regulated by short-term N-starvation and induced in long-term N-starvation.

The gene with most prominent changes in transcriptional values under N depletion was *CaNRTa*. Its ortholog in *C. canephora, Cc02_g36020*, is expressed in several tissues, with prevalence in pistil. Kanno *et al*. (2012) demonstrated that the *Arabidopsis* ortholog of this N transporter (Table 1, Figure S2) is also involved in abscisic acid transport, suggesting that this transporter may have role in several abiotic stress responses.

### Uptake of ammonium exceeds nitrate in *C. arabica* roots

**Figure 9:**
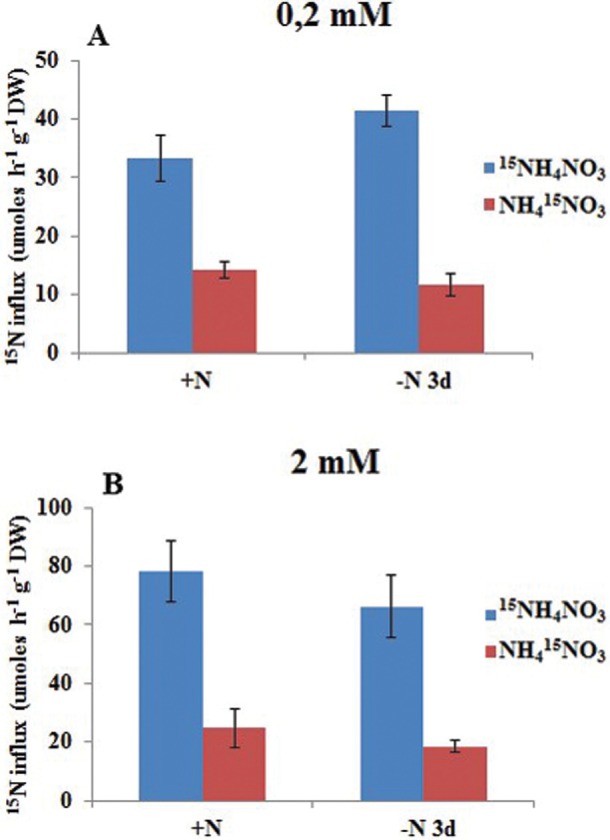
HATS (A) and LATS (B) under N starvation in *C. arabica* roots, using ammonium nitrate labeled with ^15^N. Details of the uptake experiment are described in Material and Methods.

To determine the preferential inorganic N source of coffee roots, plants were acclimated in nutrient solution under N-sufficient or N-deficient conditions and short-term ^15^N-labeled influxes with equimolar concentrations of ^15^NH_4_NO_3_ or NH_4_^15^NO_3_ were measured. For HATS activity, root ^15^N-label was measured at 0.2 mM, and the LATS activity was estimated for 2 mM of external ^15^NH_4_^+^ or ^15^NO_3_^-^ concentrations. At sufficient N supply, ^15^NH_4_^+^ uptake measured at high-affinity concentration rates exceeded that of ^15^NO_3_^-^ by 2.3-fold (Figure 9A), while in N-deficient plants, the ammonium influxes were 3.5-fold higher compared to nitrate uptake (Figure 9A). LATS activity became apparent at higher external N concentration, 2 mM ^15^NH_4_NO_3_ or NH_4_^15^NO_3_, where ^15^NH_4_^+^ influxes were 2.3-fold increased under N sufficient and 1.6-fold higher for N deficient roots in comparison to low external N supply (Figure 9A and B). By contrast, the NO_3_^-^ LATS displayed less activity, since only 1.7-fold and 1.5-fold higher ^15^NO_3_^-^ influxes were observed under ample and limited N supply, respectively (Figure 9B), when compared to HATS. In addition, at low affinity external concentrations, ^15^NH_4_^+^ influxes were 3.5 times higher than those of ^15^NO_3_^-^, independent of the N nutritional status of the plants (Figure 9B). Taken together, these results demonstrate that high and low-affinity transport systems in coffee roots are active for both inorganic N forms, and that under low external N availability, the preference for ammonium uptake over nitrate indicates that the HATS is differentially regulated. Evidence for this come from the ^15^N-labeled influxes in contrasting N supply growth conditions, in which N deficiency for three days caused an induction of 1.3-fold of HATS activity for ammonium influxes but not for nitrate uptake rates (Figure 9A). Conversely, regulation of LATS activity was absent irrespective of N form or plant N status (Figure 9B).

Physiological studies have demonstrated the presence of two high affinity transport systems for nitrate and one for ammonium in higher plants (Crawford and Glass, 1998; Loqué and von Wirén, 2004). Influx measurements in roots of several species revealed that a low capacity, constitutive active transport system is responsible for acquisition of nitrate and ammonium from low external N concentrations, and the extent of this absorption is variable among different species (Siddiqi *et al*., 1989; Serna *et al*., 1992; Wang *et al*., 1993; Kronzucker *et al*., 1997, 1998; Näsholm *et al*., 1998; Rawat *et al*., 1999; Zhuo *et al*., 1999; von Wirén *et al*., 2000; Tischner, 2000). Furthermore, for both N forms, an inducible high affinity transport system (iHATS) occurs in plants, in which HATS activity is transiently activated under N limited growth conditions and is repressed by high external N supply (Rawat *et al*., 1999; von Wirén *et al*., 2000; Nazoa *et al*., 2003; Orsel *et al*., 2006; Loqué *et al*., 2006). In addition, a key feature of the nitrate iHATS activity is that it can be rapidly induced in the presence of nitrate (Aslam *et al*., 1996; Kronzucker *et al*., 1999) although it seems to be less effective for ammonium (Loqué and von Wirén, 2004; Loqué *et al*., 2007; Lanquar *et al*., 2009).

The lack of activation of nitrate uptake by N deficient coffee roots might indicate that ammonium either causes a repression on nitrate uptake mediated by HATS, or that NO_3_-HATS is unable to be regulated under these conditions to sustain efficient nitrate acquisition in coffee roots. The inhibitory effect of ammonium on NO_3_-HATS is a common feature previously shown in roots from several species, irrespective of plant N status (Minotti *et al*., 1969; Lee and Drew, 1986; Marschner *et al*., 1991; Orsel *et al*., 2006; Robinson *et al*., 2011). In contrast, the inability to regulate nitrate iHATS under N deficiency is unknown, despite the fact that iHATS were shown to be defective in *Citrus* roots under nitrate provision or decreased NH_4_/NO_3_ ratios (Camañes *et al*., 2009). In distinction to the regulation of the nitrate HATS, the LATS for ammonium and nitrate influx appeared to be insensitive to N status in coffee roots, as previously also shown for other higher plants (Siddiqi *et al*., 1990; Wang *et al*., 1993; Rawat *et al*., 1999; Cerezo *et al*., 2000), with exception for *Arabidopsis* (Okamoto *et al*., 2003). Considering that only a few physiological conditions have been investigated, the results presented here provide initial evidence for differential regulation of HATS activity for nitrate and ammonium in coffee roots and therefore, open questions and perspectives for further investigation.

Regardless of the mechanism responsible for this effect on nitrate uptake in coffee roots, our results show that when both inorganic N sources (NH_4_^+^ and NO_3_^-^) are present in the nutrient solution, uptake of NH_4_^+^, mediated by either transport system (HATS or LATS), is favored compared to that of NO_3_^-^. This is commonly observed in several plant species and genera, including *Citrus* (Serna *et al*., 1992; Gessler *et al*., 1998; Gazzarrini *et al*., 1999; Min *et al*., 2000; Camañes *et al*., 2009), although, this situation results in greater availability of nitrate for leaching or denitrification, and further reduces the N use efficiency in coffee plants.

## Conclusions

We presented in this study a genome-wide inventory of ammonium and nitrate transporter families in *C. canephora*, taking advantage of this recently released genome. We depicted transcriptional profile and phylogenetic patterns of N transporters in this tree species, and demonstrated that *C. canephora* genomic and transcriptional patterns follow the ones observed for most eudicots. Transcriptional analysis of selected transporters in *C. arabica* roots display distinct patterns, reinforcing that each member of the *AMT* and *NRT* families has a particular role in N uptake, which is influenced by N deprivation. N-starvation demonstrated that ammonium uptake is favored over nitrate, in *C. arabica* roots. In summary, our study shows that, although nitrate transporters are prevalent compared to ammonium transporters in the *Coffea* genome, ammonium uptake is a preferential inorganic N source compared to nitrate. Additional approaches to dissect N-regulatory networks and molecular mechanisms underlying the spatial and temporal nature of the N transport response according to N demand for coffee plants are still necessary for detailed comprehension of N metabolism in coffee trees.

## Supplementary material

The following online material is available for this article:

Figure S1 - N starvation experiment

Figure S2 - Neighbor joining phylogenetic analysis of the *NRT1/PTR* family.

Figure S3 - Semi-quantitative RT-PCR analysis of *CaAMTs* and *CaNRTs*.

Table S1
*- Coffea canephora AMT1* gene family overall features.

Table S2
*- Coffea canephora AMT2* gene family overall features.

Table S3
*- Coffea canephora NRT1* gene family overall features.

Table S4
*- Coffea canephora NRT2* gene family overall features.
