# Supplementary material for: Genome-wide identification, classification and transcriptional analysis of nitrate and ammonium transporters in Coffea
Source: Genet Mol Biol. 2017 Apr 10;40(1 Suppl 1):346–59. doi: 10.1590/1678-4685-GMB-2016-0041 (PMC5452133; doi:10.1590/1678-4685-GMB-2016-0041)
Supplement: Supplementary file 1 [file 1415-4757-gmb-1678-4685-GMB-2016-0041-Suppl01.pdf]

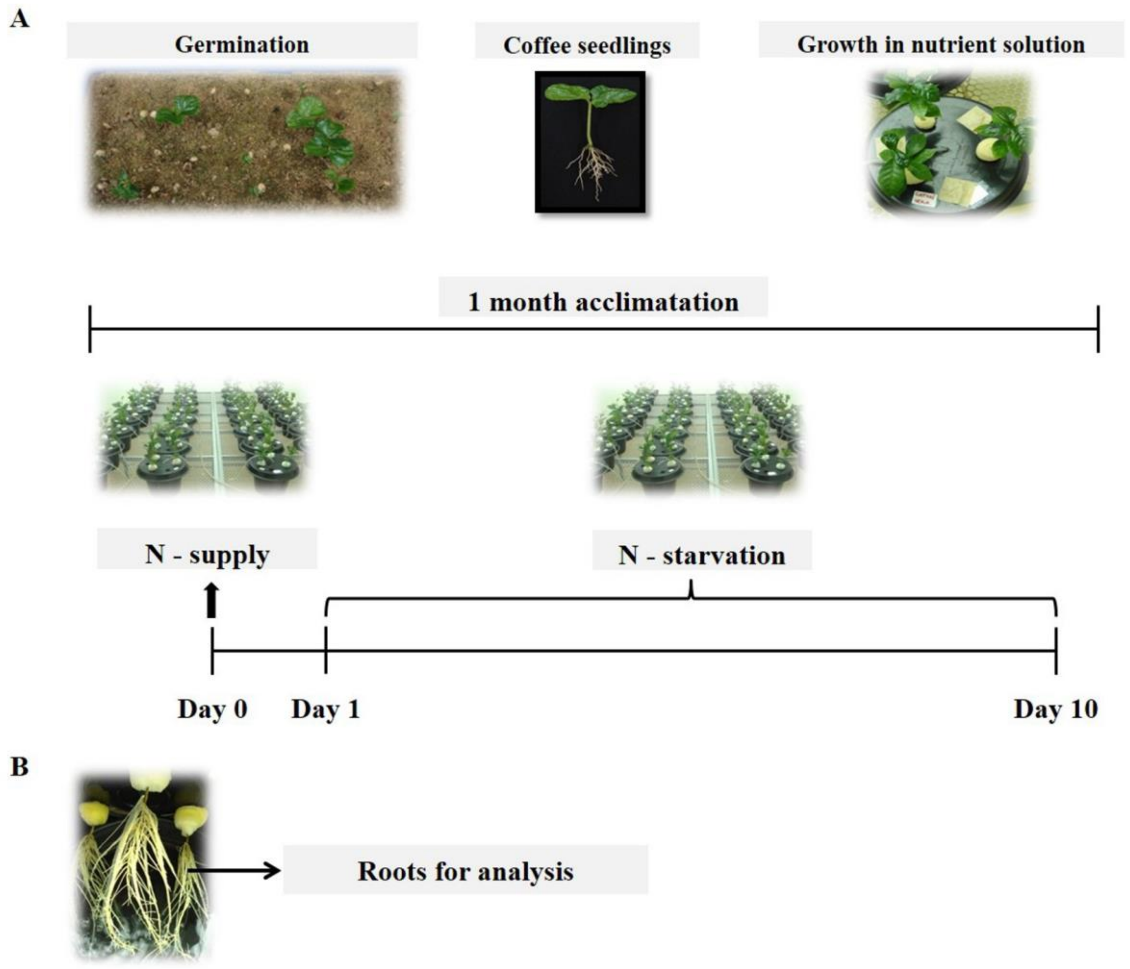

Figure S1 - N starvation experiment. **A.** Plants were germinated in sandboxes and transferred to pots with complete N supply. Three month-old plants were transferred to a nutrient solution based on Clark (1975). After acclimation, plants were harvested (Experimental Control - Day 0), and then transferred to N-free solution for ten days. **B.** For each sampling point (Day 0, Day 1 and Day 10) lateral roots were collected.
