# Supplementary material for: Genome-wide identification, classification and transcriptional analysis of nitrate and ammonium transporters in Coffea
Source: Genet Mol Biol. 2017 Apr 10;40(1 Suppl 1):346–59. doi: 10.1590/1678-4685-GMB-2016-0041 (PMC5452133; doi:10.1590/1678-4685-GMB-2016-0041)
Supplement: Supplementary file 2 [file 1415-4757-gmb-1678-4685-GMB-2016-0041-Suppl04.pdf]

Table S1 - *Coffea canephora AMT1* gene family overall features: Gene name, subcellular localization, number of transmembrane domains (TM) and in silico expression profile (RPKM).

Name	Subcellular localization	TM	*In silico* expression profile (RPKM)					
			Root	Stamen	Pistil	Leaf	Perisperm	Endosperm
Cc01_g14140*	Endoplasmic reticulum	9	2.5	2	13.5	0.2	1	0
Cc01_g17670	Endoplasmic reticulum	10	93.8	0.1	0.1	0.2	0.2	0
Cc03_g06810*	Golgi apparatus	11	22.3	63.9	46.4	37.7	150.1	23.8
Cc09_g03020	Endoplasmic reticulum	11	89.2	0.2	0.4	0.7	0	0

* *Coffea arabica* orthologs: Cc03_g06810 = *CaAMTa*; Cc01_g14140 = *CaAMTb*.
