# Supplementary material for: Genome-wide identification, classification and transcriptional analysis of nitrate and ammonium transporters in Coffea
Source: Genet Mol Biol. 2017 Apr 10;40(1 Suppl 1):346–59. doi: 10.1590/1678-4685-GMB-2016-0041 (PMC5452133; doi:10.1590/1678-4685-GMB-2016-0041)
Supplement: Supplementary file 3 [file 1415-4757-gmb-1678-4685-GMB-2016-0041-Suppl05.pdf]

**Table S2 -** *Coffea canephora AMT2* gene family overall features: Gene name, subcellular localization, number of transmembrane domains (TM) and in silico expression profile (RPKM).

| Name | Subcellular localization | TM | *In silico* expression profile (RPKM) | | | | | |
|---|---|---|---|---|---|---|---|---|
| | | | Root | Stamen | Pistil | Leaf | Perisperm | Endosperm |
| Cc02_g30580 | Cytoplasmic | 11 | 2.5 | 0 | 0 | 0 | 0 | 0 |
| Cc07_g11400 | Golgi apparatus | 11 | 0.5 | 1 | 0.1 | 0.1 | 0 | 0 |
| Cc07_g19360* | Cytoplasmic | 11 | 20 | 2.3 | 0.5 | 3.9 | 3.2 | 0 |
| Cc11_g01840 | Cytoplasmic | 11 | 1.2 | 0.2 | 2.1 | 1.2 | 0.4 | 0.3 |

* *Coffea arabica* ortholog: Cc07_g19360 = *CaAMTc*
