# Supplementary material for: Genome-wide identification, classification and transcriptional analysis of nitrate and ammonium transporters in Coffea
Source: Genet Mol Biol. 2017 Apr 10;40(1 Suppl 1):346–59. doi: 10.1590/1678-4685-GMB-2016-0041 (PMC5452133; doi:10.1590/1678-4685-GMB-2016-0041)
Supplement: Supplementary file 4 [file 1415-4757-gmb-1678-4685-GMB-2016-0041-Suppl06.pdf]

Table S3. *Coffea canephora NRT1* gene family overall features: Gene name, subcellular localization, number of transmembrane domains (TM) and *in silico* expression profile (RPKM).

Name	Subcellular localization	TM	*In silico* expression profile (RPKM)					
			Root	Stamen	Pistil	Leaf	Perisperm	Endosperm
Cc00_g26240	Cytoplasmic	12	2.9	0.3	0.2	9.1	0.5	0.5
Cc00_g29690	Plasma membrane	11	33.5	1.3	7.3	8.5	10.4	8.4
Cc00_g31780	Extracellular	10	65.1	0.8	2.5	10.4	3.3	3.8
Cc01_g05330	Peroxisome	10	54.4	0.1	0	0	0	0
Cc01_g06540	Endoplasmic reticulum	11	0	0	0	0	0	0
Cc01_g06870	Golgi apparatus	12	0.5	0.7	0.1	0.1	0.2	0
Cc01_g11730	Plasma membrane	12	13.5	1.7	0.2	0.6	0.7	0
Cc01_g11750	Plasma membrane	12	26.6	0.1	0.1	0	0	0
Cc01_g11760	Plasma membrane	10	0.2	0.1	0	0	0	0
Cc01_g11770	Plasma membrane	10	0.1	0.1	0.1	0.1	1.3	0.9
Cc01_g11780	Plasma membrane	9	5.8	0.3	6.2	0.9	1.9	2.4
Cc01_g13830	Plasma membrane	10	43.5	1.7	5.6	24.7	20.9	32.7
Cc01_g13840	Plasma membrane	10	0.1	0.1	5	8.8	0	0
Cc01_g13850	Cytoplasmic	10	20.2	1.2	0.1	6.7	71	0
Cc01_g17970	Cytoplasmic	11	0.2	0	0	0	0	0
Cc02_g05650	Cytoplasmic	10	20.9	3.7	62.3	22.9	95.5	43.2
Cc02_g07300	Cytoplasmic	10	0.6	0.2	0	0	0	0
Cc02_g16010	Golgi apparatus	10	0.1	0	0	0	0	0
Cc02_g23600	Cytoplasmic	12	6.8	0.2	24.5	15.5	1.1	1.8
Cc02_g24100	Cytoplasmic	12	5.7	0.7	1.4	2.7	0.4	0.7
Cc02_g25980	Golgi apparatus	12	5.8	11.5	0.1	0.1	0.3	0.4
Cc02_g36020*	Golgi apparatus	12	26.7	70.3	373	49.2	49	3.3
Cc03_g05950	Cytoplasmic	11	4.6	1	0.1	0.4	1.2	0
Cc03_g05960	Cytoplasmic	11	70.4	0.2	0.1	0.2	15.7	0
Cc03_g14140	Cytoplasmic	9	0.1	0.4	0	0	0	0
Cc04_g02930	Cytoplasmic	11	10.2	3.3	0.2	13.3	4.5	0
Cc04_g02940	Golgi apparatus	10	1.5	0.2	0.5	1.1	0.4	0.3
Cc04_g15590	Plasma membrane	12	0.2	22.9	0.7	0.1	0.7	0
Cc04_g15710	Plasma membrane	11	52.2	0.1	0	0	0	0
Cc04_g15720	Cytoplasmic	12	0.5	0.2	0.2	2.4	40	0
Cc04_g15730	Cytoplasmic	11	14.5	2.4	26.1	3.1	0.2	0.2
Cc04_g15760	Cytoplasmic	8	2.2	1.9	1.3	0.3	5.5	2.6
Cc04_g15770*	Golgi apparatus	10	13.4	8.7	667.2	38.7	84.6	99.9
Cc06_g02110	Plasma membrane	8	55.3	1.4	5.8	0.6	9.4	0
Cc06_g08580*	Cytoplasmic	11	12.3	1.3	260.1	4.7	15.9	0
Cc06_g10000	Golgi apparatus	9	0	0	0	0	0	0
Cc06_g20220	Golgi apparatus	9	20.9	7.4	0.1	0	0	0
Cc06_g20230	Peroxisome	8	18	1.8	2.7	2.5	1.6	1
Cc07_g02690	Golgi apparatus	11	60	1.3	0.5	2.1	9.6	2.3
Cc08_g02340	Golgi apparatus	10	20.7	4.6	0.1	0.7	7.5	9.4
Cc08_g09260	Cytoplasmic	9	0.7	0.9	10.1	0.9	3.1	15.1
Cc08_g09340	Golgi apparatus	9	0.1	0.3	4.4	11	3.8	13.5
Cc08_g09360	Plasma membrane	11	0.3	7.4	8	34.9	10.6	6.8
Cc08_g09370	Plasma membrane	9	0.4	3.8	5.2	15	48.3	7.4
Cc08_g12800	Peroxisome	11	96.2	94.7	17.4	2.7	29.4	4
Cc10_g02250	Cytoplasmic	12	0.5	0.5	1.8	0.2	1.3	0.1

Name	Subcellular localization	TM	*In silico* expression profile (RPKM)					
			**Root**	**Stamen**	**Pistil**	**Leaf**	**Perisperm**	**Endosperm**
Cc10_g03760	Plasma membrane	10	0.1	0.1	0.8	0.5	0.4	2.1
Cc10_g08680	Golgi apparatus	11	3.9	0.1	0.1	51.4	2.9	0
Cc10_g09750	Cytoplasmic	12	2.1	1.7	4.3	52.4	60.5	0.4
Cc10_g09920	Cytoplasmic	10	17.1	30.3	17.9	12.6	14.1	21.1
Cc10_g09930	Cytoplasmic	10	21	11	7.7	4.3	22.8	21
Cc10_g12560	Cytoplasmic	11	4.2	2.8	11.6	1.7	21.3	11.6
Cc10_g16140	Cytoplasmic	12	14.9	0.1	4.5	1.6	2.3	0.5
Cc11_g05180	Cytoplasmic	10	61.8	48.9	51.3	39.5	42.2	36.7
Cc11_g13530	Plasma membrane	11	30.3	0.9	124.4	5.2	6.9	0.2
Cc11_g13590	Cytoplasmic	12	27.9	113.1	471.6	119.6	31.1	246.7
Cc11_g14930	Cytoplasmic	12	0.6	1.8	25.8	36.5	8.6	17.2

* *Coffea arabica* orthologs: Cc02_g36020 = *CaNRTa*; Cc06_g08580 = *CaNRTb*; Cc04_g15770 = *CaNRTc*.
