# Supplementary material for: Genome-wide identification, classification and transcriptional analysis of nitrate and ammonium transporters in Coffea
Source: Genet Mol Biol. 2017 Apr 10;40(1 Suppl 1):346–59. doi: 10.1590/1678-4685-GMB-2016-0041 (PMC5452133; doi:10.1590/1678-4685-GMB-2016-0041)
Supplement: Supplementary file 5 [file 1415-4757-gmb-1678-4685-GMB-2016-0041-Suppl02.pdf]

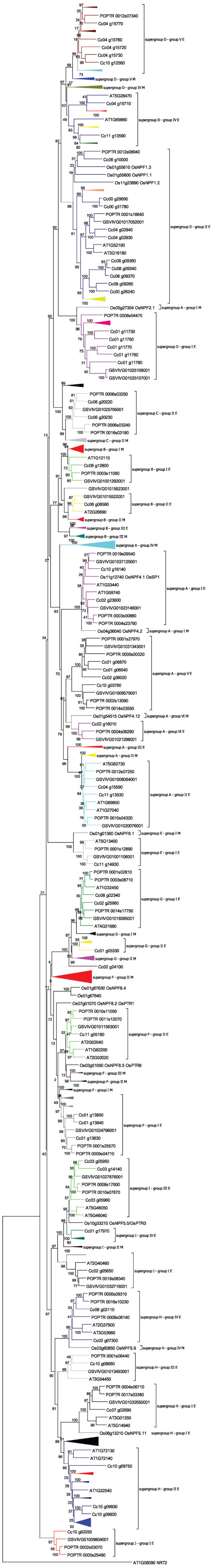

Figure S2 - Neighbor joining phylogenetic analysis of the *NRT1/PTR* family. The tree was rooted using an *A. thaliana NRT2* gene as an outgroup. Percent bootstrap values from 1,000 replicates are given. All *C. canephora* genes are placed in clades with >50% of bootstrap support. Taxonomic groups are differentially colored based in supergroups identified for monocots and eudicots. Accession numbers are shown. Codes were retrieved from the Coffee Genome Hub for C. *canephora* sequences and Phytozome for all other species. Phylogenetic groups were based in von Wittgenstein *et al.* (2014).
