# Supplementary material for: Genome-wide identification, classification and transcriptional analysis of nitrate and ammonium transporters in Coffea
Source: Genet Mol Biol. 2017 Apr 10;40(1 Suppl 1):346–59. doi: 10.1590/1678-4685-GMB-2016-0041 (PMC5452133; doi:10.1590/1678-4685-GMB-2016-0041)
Supplement: Supplementary file 6 [file 1415-4757-gmb-1678-4685-GMB-2016-0041-Suppl07.pdf]

Table S4 - *Coffea canephora NRT2* gene family overall features: Gene name, subcellular localization, number of transmembrane domains (TM) and in silico expression profile (RPKM).

Name	Subcellular localization	TM	*In silico* expression profile (RPKM)					
			Root	Stamen	Pistil	Leaf	Perisperm	Endosperm
Cc01_g10620	Cytoplasmic	11	10.8	0.9	0	0	0	0
Cc01_g10640	Peroxisome	11	111.8	0.1	0	0	0	0
Cc11_g15480	Peroxisome	11	8.4	0.1	15.4	0.4	5.1	6.7
