# Supplementary material for: Genome-wide identification, classification and transcriptional analysis of nitrate and ammonium transporters in Coffea
Source: Genet Mol Biol. 2017 Apr 10;40(1 Suppl 1):346–59. doi: 10.1590/1678-4685-GMB-2016-0041 (PMC5452133; doi:10.1590/1678-4685-GMB-2016-0041)
Supplement: Supplementary file 7 [file 1415-4757-gmb-1678-4685-GMB-2016-0041-Suppl03.pdf]

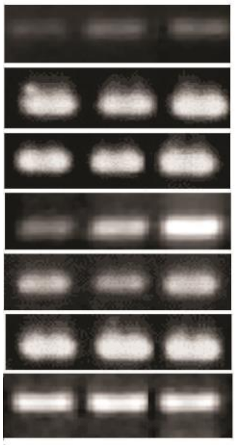

Figure S3 - Semi-quantitative RT-PCR analysis of *CaAMTs* and *CaNRTs* using cDNA from *C. arabica* roots under N – starvation (details are described in Materials and Methods, Figure 7 and Figure 8).
